# Shen Song Yang Xin Capsule Combined with Antiarrhythmic Drugs, a New Integrative Medicine Therapy, for the Treatment of Frequent Premature Ventricular Contractions (FPVC): A Meta-Analysis of Randomized Controlled Trials

**DOI:** 10.1155/2014/976713

**Published:** 2014-03-18

**Authors:** Jie Wang, Jun Li, Bo Feng

**Affiliations:** Department of Cardiology, Guang'anmen Hospital, China Academy of Chinese Medical Sciences, Beixiange No. 5, Xicheng District, Beijing 100053, China

## Abstract

*Objective*. To evaluate the beneficial and adverse effects of Shen Song Yang Xin Capsule (SSYX Capsule) combined with antiarrhythmic drugs for the treatment of frequent premature ventricular contractions (FPVC). *Methods*. Seven electronic databases were searched to retrieve any potential randomized controlled trials (RCTs) designed to evaluate the clinical efficacy of SSYX Capsule combined with Antiarrhythmic Drugs for FPVC reported in any language, with total effect for FPVC and number of ventricular premature contraction as the main outcome measure. The methodological quality of the included studies was assessed using criteria from the Cochrane Handbook for Systematic Review of Interventions, Version 5.1.0, and analysed using RevMan 5.1.0 software. *Results*. Sixteen RCTs of SSYX Capsule were included. The methodological quality of the trials was generally evaluated as low. The results of meta-analysis showed that SSYX Capsule combined with antiarrhythmic drugs was more effective in total effect for FPVC and number of ventricular premature contraction compared with Antiarrhythmic Drugs in patients with FPVC or FPVC complicated by other diseases. Ten of the trials reported adverse events, indicating that the safety of SSYX Capsule is still uncertain. *Conclusions*. There is some but weak evidence about SSYX Capsule combined with antiarrhythmic drugs appearing to be more effective in total effect for FPVC and number of ventricular premature contraction in patients with FPVC and its complications.

## 1. Introduction

Frequent premature ventricular complexes (PVCs) are a common occurrence in clinical practice. It is responsible for considerable morbidity and mortality. It may cause haemodynamic deterioration and reversible left ventricular (LV) dysfunction and can act as markers to other cardiac diseases such as cardiomyopathy and ischemic heart disease [1]. Recent research demonstrated that the quantitative additional mortality risk on exercise test (ET) presented by frequent PVCs is similar to ischemia [2]. Clinical presentation of PVCs may be quite variable, ranging from an incidental finding on electrocardiogram (ECG) to congestive heart failure (CHF) [3]. For patients with symptoms that can be attributed to PVCs, the medical therapy of beta blockers or class I or III antiarrhythmic agents is effective and recommended. However, some patients cannot tolerate medical therapy. Currently, radiofrequency catheter ablation (RFCA) been increasingly used of symptomatic PVCs has been reported to be a safe and efective treatment option [4, 5].

With increasing popularity of complementary and alternative medicine among patients with PVCs, TCM (Traditional Chinese Medicine) is becoming more and more frequently used both in China and western countries [6]. Shen Song Yang Xin Capsule (SSYX Capsule) is a widely used Chinese medicine with antiarrhythmic effect. Many clinical researches showed that it can effectively improve symptoms of heart palpitations, chest tightness, shortness of breath, insomnia, fatigue, and so on [7–9]. Mechanism of antiarrhythmic effect is mainly due to blockade effect of different ion channels [10, 11]. Although there has been much research on SSYX Capsule combined with Western medicine for the treatment of FPVC, there is no critically appraised evidence such as systematic reviews or meta-analyses on potential benefits. This report aims to evaluate the beneficial and adverse effects of SSYX Capsule combined with antiarrhythmic drugs for the treatment of FPVC on total effect for FPVC and number of ventricular premature contraction.

## 2. Materials and Methods

### 2.1. Database and Search Strategies

We selected all the clinical trials about SSYX Capsule combined with antiarrhythmic drugs for treatment of FPVC in the Chinese National Knowledge Infrastructure (CNKI), the Chinese Biomedical Literature Database (CBM), the Chinese Scientific Journal Database (VIP), PubMed, and the Cochrane Central Register of Controlled Trials in the Cochrane Library (May 2013). We also searched the reference list of retrieved papers. Databases in Chinese were searched to retrieve the maximum possible number of trials of SSYX Capsule for FPVC because it is mainly used in China. The following search terms were used individually or combined: “frequent premature ventricular contractions,” “frequent premature ventricular beats,” “Shen Song Yang Xin Capsule,” “combined with,” “controlled clinical trial,” “clinical trial,” and “Randomized Controlled Trials.”

### 2.2. Inclusion and Exclusion Criteria

All randomized controlled trials (RCTs) of patients with FPVC that studied prescriptions based on SSYX Capsule combined with antiarrhythmic drugs compared with antiarrhythmic drugs were included. There were no restrictions on language, population characteristics, and publication type. The primary outcome measure was total effect for FPVC and number of ventricular premature contractions. Duplicated publications reporting the same groups of participants were excluded.

### 2.3. Data Extraction and Quality Assessment

Two authors (Jun Li and Bo Feng) conducted the literature searching, study selection, and data extraction independently. The extracted data included the title of the study, authors, year of publication, article source, study size, total number of cases (Treatment/Control), diagnosis standard, details of methodological information, and treatment course, and clinical standards as well as the details of the control interventions, outcomes, and adverse effects for each study. Disagreement was resolved by discussion and reached consensus through a third party (Jie Wang). The methodological quality of included trials was assessed according to the Cochrane Handbook for Systematic Review of Interventions, Version 5.1.0 [12]. The assessment used the following 6 criteria, including random sequence generation (selection bias), allocation concealment (selection bias), blinding of participants and personnel (performance bias), blinding of outcome assessment (detection bias), incomplete outcome data (attrition bias), selective reporting (reporting bias), and other bias.

### 2.4. Data Synthesis

RevMan 5.1.0 software provided by the Cochrane Collaboration was used for data analyses. Dichotomous data were expressed as relative risk (RR) and continuous outcomes were presented as weighted mean difference (WMD), while 95% confidence intervals (CI) were calculated for both. Meta-analysis was performed if the intervention, control, and outcomes were the same or similar. The statistical heterogeneity was presented as significant when the* I* square (*I* ^2^) value exceeded 50% or *P* < 0.1. In the absence of significant heterogeneity, we pooled data using fixed effects model (*I* ^2^ < 50%); otherwise we used random effects model (*I* ^2^ > 50%).

## 3. Results

### 3.1. Description of the Included Trials

**Figure 1:**
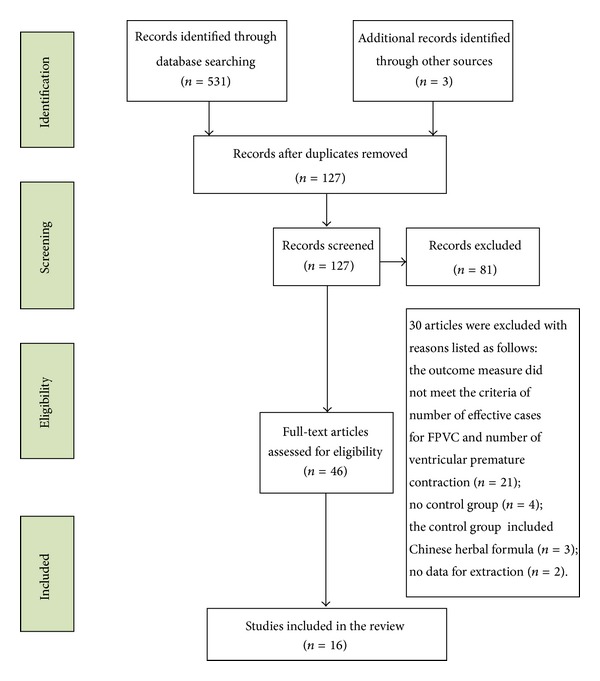
Study selection process.

After the primary search of the five databases, 534 potentially relevant trials were screened out from electronic and manual searches. The majority were excluded because duplicates were removed and some included irrelevant titles and abstracts. Only 127 studies were retrieved. Following review of the titles and abstracts, several studies were excluded, and only 46 studies remained. 21 trials did not use the criteria of total effect for FPVC and number of ventricular premature contractions as the outcome measure. 4 trials have no control group. 3 clinical trials included Chinese herbal formula as the control group and 2 trials had no data for extraction. In the end, sixteen RCTs were to be reviewed [13–28]. The study selection process is summarised in a flow chart (Figure 1). All of the trials were conducted in China and published in Chinese. The characteristics of the sixteen RCTs are summarised in Table 1.

The sixteen RCTs involved a total number of 1115 patients with FPVC. One trial [15] study FPVC with sick sinus syndrome, four trials [13, 20, 26, 28] study elderly FPVC and other studies FPVC without detailed information. Of those, one [15] used an international consensus on nomenclature and classification of FPVC developed by ACC/AHA/ESC 2008 Guidelines for the Management of Patients with FPVC (ACC/AHA/HRS 2008). Two trials demonstrated that patients with FPVC diagnosis were more than 720/24 h [13, 16]. Four trials of FPVC diagnosis were more than 30/h [17, 19, 24, 26]. One trial used diagnosis criteria of more than 5/min [18]. One trial used diagnosis criteria of more than 1000/24 h. One trial used diagnosis criteria of more than 7200/24 h. The rest of the trials [14, 21, 22, 25, 27, 28] only demonstrated patients with FPVC diagnosis by electrocardiogram and 24-hour Holter without detailed information.

The interventions of all sixteen trials [13–28] included SSYX Capsule combined with antiarrhythmic drugs. One control used conventional therapy with antiarrhythmic drugs with no detailed information [15]. The other controls were antiarrhythmic drugs alone. The total treatment duration ranged from 1 month to 6 months. Thirteen trials [13, 16, 19, 21] specified clinical standards of FPVC by “Clinical research guideline of medicine for cardiovascular system by Ministry of Public Health (1998).” The clinical standards of FPVC by “Clinical research guideline of medicine for cardiovascular system by Ministry of Public Health (1998)” were described as follows. Marked effective result: ventricular premature contraction diminished or decreased more than 90% in 24-hour Holter. Chest distress and palpitation symptoms disappeared or improved. Effective result: ventricular premature contraction diminished or decreased more than 50% in 24-hour Holter. Chest distress and palpitation symptoms partially disappeared or improved. Ineffective result: ventricular premature contraction diminished or decreased less than 50% in 24-hour Holter. Chest distress and palpitation symptoms had no improvement or aggravated.

All the sixteen trials [13–28] used the total effect for FPVC as the main outcome measure, and five of the sixteen trials [15, 17, 19, 20, 24] used the number of ventricular premature contraction as the main outcome measure. Ten of the included trials [13–15, 17, 20, 22–24, 26, 27] described adverse effects in detail.

### 3.2. Methodological Quality of the Included Trials

The majority of the included trials were assessed to be of general poor methodological quality according to the predefined quality assessment criteria (As shown in Table 2). The randomized allocation of participants was mentioned in all trials. However, only one trial stated the methods for sequence generation by random number table [16]. None of the trials calculated an estimation of the pretrial sample size; there may be insufficient power to ensure appropriate estimation of the therapeutic effect. Allocation concealment and blinding of outcome assessment were not mentioned in all trials.

### 3.3. Effects of the Interventions

#### 3.3.1. Effective Frequency Number for FPVC

**Figure 2:**
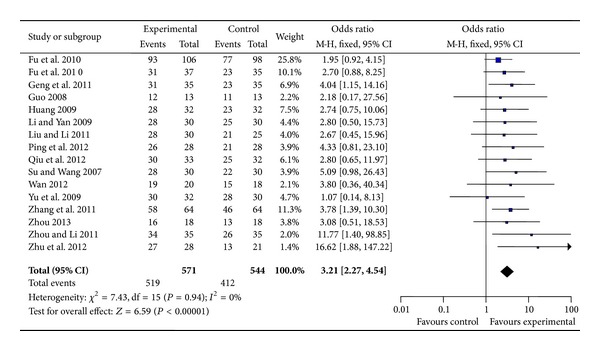
Analysis of total effect for FPVC. Forest plot of comparison: SSYX Capsule combined with antiarrhythmic drug group versus antiarrhythmic drug group.

All the sixteen trials [13–28] used the total effect for FPVC as an outcome measure. These sixteen trials compared the combination of SSYX Capsule plus antiarrhythmic drugs with antiarrhythmic drugs alone. Trial results for the sixteen independent trials were homogeneous, *χ* ^2^ = 7.43, df = 15, (*P* = 0.94); *I* ^2^ = 0%, requiring the use of the fixed effects model for statistical analysis. The effective frequency number after SSYX Capsule combined with antiarrhythmic drugs was more than antiarrhythmic drug treatment. The meta-analysis demonstrated a significant difference between the two groups. (MD: 3.21 [2.27, 4.54]; *P* < 0.00001) (Figure 2).

#### 3.3.2. Number of Ventricular Premature Contractions

**Figure 3:**
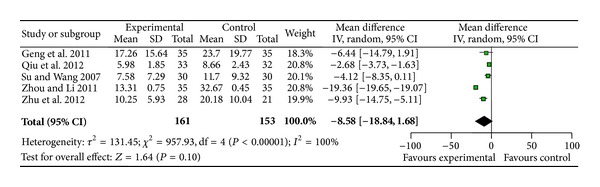
Analysis of number of ventricular premature contractions. Forest plot of comparison: SSYX Capsule combined with antiarrhythmic drug group versus antiarrhythmic drug group.

Five trials [15, 17, 19, 20, 24] used the number of ventricular premature contractions as an outcome measure. Trial results for the seven independent trials were not homogeneous, *χ* ^2^ = 957.93, df = 4, (*P* < 0.00001); *I* ^2^ = 100%, requiring the use of the random effects model for statistical analysis. The number of ventricular premature contractions in the SSYX Capsule combined with antiarrhythmic drug group was less than that of antiarrhythmic drug group, but the meta-analysis did not show significant beneficial effect in the combination group compared with the antiarrhythmic drug group (RR −8.58; 95% CI [−18.84, 1.68]; *P* = 0.10) (Figure 3).

### 3.4. Subgroup Analysis and Publication Bias

The number of trials was too small to conduct analysis of subgroup and publication bias.

### 3.5. Adverse Effects

Ten out of the included trials [13–15, 17, 20, 22–24, 26, 27] described adverse effects in detail. All the ten cases mentioned specific symptoms, including sleepiness, nausea, loss of appetite, stomach discomfort, and sinus bradycardia, in the SSYX Capsule combined with antiarrhythmic drugs group and I atrioventricular block, II atrioventricular block, sinus bradycardia, abdominal distension, and so on in the antiarrhythmic drugs group. These side effects may be related to the adverse effect of amiodarone. Eight trials [13, 14, 17, 20, 22–24, 26] mentioned adverse effects in both groups and mentioned specific symptoms. In the experimental group, one case of sleepiness was reported, seven cases of nausea, one case of loss of appetite, twenty cases of stomach discomfort, and seven cases of dizziness and one case demonstrated adverse effect of sinus bradycardia. In the controlled group, one case reported adverse effect of I atrioventricular block, one case I atrioventricular block and* U* wave, thirteen cases nausea, abdominal distension, and stomach discomfort, ten cases sinus bradycardia, of which three cases were serious, two cases II atrioventricular block, two cases low blood pressure, four cases liver function damage, seven cases dizziness, and three cases Q-T interval prolongation and one case showed adverse effect of Q-T interval prolongation combined with paroxysmal ventricular tachycardia. One trial [15] reported adverse effects in the SSYX Capsule combined with antiarrhythmic drugs group of mildly stomach discomfort in four cases. One trial [27] reported adverse effects in the antiarrhythmic drugs group of sinus bradycardia in three cases. In general, the incidence of adverse reactions was lower in the treatment group compared with the control group.

## 4. Discussion

PVCs is common clinical heart disease. It can present with a wide spectrum of symptoms in patients with and without structural heart disease. In patients with structural heart disease, they may cause or aggravate the disease process, or be a consequence of the disease process itself. The main symptom of this disease in clinic is palpitation, which can induce feeling of heart pounding in patients or the feeling of dizziness and anxiety and even syncope. This disease not only cause abnormal work and life in patients, and even endanger the safety of life seriously.

Conventional medicine therapy can inhibit the deterioration of clinical symptoms of the patients in a short period of time but cannot reduce the physical and psychological suffering of patients fundamentally. TCM has long history and abundant experiences in treatment of clinical manifestations frequently reported by patients with frequent premature ventricular contractions. Currently, with wide acceptance of integrative medicine (IM) for primary health care worldwide, more and more people with CVDs select CAM/TCM to maintain their health for more efficacy and less adverse effect [29, 30]. Although there are still some problems in this domain, evidence based IM is warranted in further researches.

This paper included 16 randomized trials and a total of 1115 participants. Shen Song Yang Xin Capsule combined with antiarrhythmic drugs, a new integrative medicine therapy, showed significant benefit on outcome of total effect and improving symptoms and signs as compared with conventional treatment for FPVC. However, due to the low-quality methodology and potential publication bias, a definite conclusion of the beneficial effectiveness of SSYX Capsule combined with antiarrhythmic drugs in treating FPVC could not be drawn. In addition, Shen Song Yang Xin Capsule is a traditional Chinese medicine. It should therefore be used under the guidance of TCM theory of “syndrome differentiation and treatment” [31–33]. Also, more guidance on the theory of traditional Chinese medicine is needed in the future.

## 5. Conclusion

There is encouraging evidence of the effect of SSYX Capsule combined with antiarrhythmic drugs on total effect for FPVC and number of ventricular premature contractions with FPVC. Due to the poor quality of experimental design and methodology, the evidence remains weak. More rigorous RCTs with strong design and high methodological quality will be needed to present a high level of evidence for the effectiveness of SSYX Capsule in treating FPVC.

## Figures and Tables

**Table 1:** Characteristics and methodological quality of the included studies.

Study	Sample size (treatment/control)	Diagnosis standard	Complications	Intervention	Control	Treatment course (month)	Clinical standards	Outcome measure
Huang 2009 [13]	64 (32/32)	Diagnosis criteria for FPVC (>720/24 h)	Elderly FPVC	SSYX Capsule 1.6 g tid + Control	Amiodarone 200 mg, tid for 4 d; 200 mg, bid for 3 d; 200 mg, qd for 14 d; 100 mg, qd for maintenance treatment	1	Clinical research guideline of medicine for cardiovascular system by Ministry of Public Health (1998)	Total effect for FPVC; ADR
Guo 2008 [14]	26 (13/13)	Diagnosis criteria for FPVC (unclear)	FPVC	SSYX Capsule 1.6 g tid + Control	Amiodarone 200 mg, tid for 7 d; 200 mg, bid for 7 d; 200 mg, qd for maintenance treatment	3	Clinical research guideline of medicine for cardiovascular system by Ministry of Public Health (1998)	Total effect for FPVC; ADR
Zhu et al. 2012 [15]	49 (28/21)	ACC/AHA/HRS (2008)	Sick Sinus Syndrome and FPVC	SSYX Capsule 1.6 g tid + Control	Conventional therapy with antiarrhythmic drugs (no detailed information)	2	Clinical research guideline of medicine for cardiovascular system by Ministry of Public Health (1998)	Total effect for FPVC; Number of Ventricular Premature Beat; ADR
Ping et al. 2012 [16]	56 (28/28)	Diagnosis criteria for FPVC (>720/24 h)	FPVC	SSYX Capsule 1.6 g tid + Control	Amiodarone 200 mg, tid for 7 d; 200 mg, bid for 14 d; 200 mg, qd for maintenance treatment	4	Clinical research guideline of medicine for cardiovascular system by Ministry of Public Health (1998)	Total effect for FPVC
Su and Wang 2007 [17]	60 (30/30)	Diagnosis criteria for FPVC (>30/h)	FPVC	SSYX Capsule 1.6 g tid + Control	Mexiletine 12.5 mg, bid	4	Clinical research guideline of medicine for cardiovascular system by Ministry of Public Health (1998)	Total effect for FPVC; Number of Ventricular Premature Beat; ADR
Yu et al. 2009 [18]	62 (32/30)	Diagnosis criteria for FPVC (>5/min)	FPVC	SSYX Capsule 1.6 g tid + Control	Metoprolol 12.5 mg, bid	2	Clinical research guideline of medicine for cardiovascular system by Ministry of Public Health (1998)	Total effect for FPVC
Zhou and Li 2011 [19]	70 (35/35)	Diagnosis criteria for FPVC (>30/h)	FPVC	SSYX Capsule 1.6 g tid + Control	Metoprolol 12.5 mg, bid	1	24 h Holter	Total effect for FPVC; Number of Ventricular Premature Beat
Geng et al. 2011 [20]	70 (35/35)	Diagnosis criteria for FPVC (>1000/24 h)	Elderly FPVC	SSYX Capsule 1.6 g tid + Control	Metoprolol 12.5 mg, bid	4	Clinical research guideline of medicine for cardiovascular system by Ministry of Public Health (1998)	Total effect for FPVC; Number of Ventricular Premature Beat; ADR
Zhou 2013 [21]	36 (18/18)	Diagnosis criteria for FPVC (unclear)	FPVC	SSYX Capsule 1.6 g tid + Control	Metoprolol 12.5 mg, bid	2	24 h Holter	Total effect for FPVC
Wan 2012 [22]	38 (20/18)	Diagnosis criteria for FPVC (unclear)	FPVC	SSYX Capsule 1.6 g tid + Control	Metoprolol 12.5 mg, bid	4	24 h Holter	Total effect for FPVC; ADR
Fu et al. 2010 [23]	204 (106/98)	Diagnosis criteria for FPVC (7200/24 h)	FPVC	SSYX Capsule 1.6 g tid + Control	Amiodarone 200 mg, tid for 3 d; 100 mg, bid for 3 d; 100 mg, qd for maintenance treatment	6	Clinical research guideline of medicine for cardiovascular system by Ministry of Public Health (1998)	Total effect for FPVC; ADR
Qiu et al. 2012 [24]	65 (33/32)	Diagnosis criteria for FPVC (>30/h)	FPVC	SSYX Capsule 1.6 g tid + Control	Metoprolol 12.5 mg, bid	2	Clinical research guideline of medicine for cardiovascular system by Ministry of Public Health (1998)	Total effect for FPVC; Number of Ventricular Premature Beat; ADR
Li and Yan 2009 [25]	60 (30/30)	Diagnosis criteria for FPVC (unclear)	FPVC	SSYX Capsule 1.6 g tid + Control	Propafenone 150 mg, tid	3	Clinical research guideline of medicine for cardiovascular system by Ministry of Public Health (1998)	Total effect for FPVC
Fu et al. 2010 [26]	72 (37/35)	Diagnosis criteria for FPVC (>30/h)	Elderly FPVC	SSYX Capsule 1.6 g tid + Control	Amiodarone 200 mg, tid for 7 d; 200 mg, bid for 14 d; 200 mg, qd for 21 d; 100–200 mg, qd for maintenance treatment	4	Clinical research guideline of medicine for cardiovascular system by Ministry of Public Health (1998)	Total effect for FPVC; ADR
Zhang et al. 2011 [27]	128 (64/64)	Diagnosis criteria for FPVC (unclear)	FPVC	SSYX Capsule 1.6 g tid + Control	Metoprolol 12.5 mg, bid	4	Clinical research guideline of medicine for cardiovascular system by Ministry of Public Health (1998)	Total effect for FPVC; ADR
Liu and Li 2011 [28]	55 (30/25)	Diagnosis criteria for FPVC (unclear)	Elderly FPVC	SSYX Capsule 1.6 g tid + Control	Propafenone 100–150 mg, tid	2	Clinical research guideline of medicine for cardiovascular system by Ministry of Public Health (1998)	Total effect for FPVC

**Table 2:** Quality assessment of included randomized controlled trials.

Included trials	Random sequence generation	Allocation concealment	Blinding of participants and personnel	Blinding of outcome assessment	Incomplete outcome data	Selective reporting	Other sources of bias
Huang 2009 [13]	Unclear	Unclear	Unclear	Unclear	No	No	Unclear
Guo 2008 [14]	Unclear	Unclear	Unclear	Unclear	No	No	Unclear
Zhu et al. 2012 [15]	Unclear	Unclear	Unclear	Unclear	No	No	Unclear
Ping et al. 2012 [16]	Table of random number	Unclear	Unclear	Unclear	No	No	Unclear
Su and Wang 2007 [17]	Unclear	Unclear	Unclear	Unclear	No	No	Unclear
Yu et al. 2009 [18]	Unclear	Unclear	Unclear	Unclear	No	No	Unclear
Zhou and Li 2011 [19]	Unclear	Unclear	Unclear	Unclear	No	No	Unclear
Geng et al. 2011 [20]	Unclear	Unclear	Unclear	Unclear	No	No	Unclear
Zhou 2013 [21]	Unclear	Unclear	Unclear	Unclear	No	No	Unclear
Wan 2012 [22]	Unclear	Unclear	Unclear	Unclear	No	No	Unclear
Fu et al. 2010 [23]	Unclear	Unclear	Unclear	Unclear	No	No	Unclear
Qiu et al. 2012 [24]	Unclear	Unclear	Unclear	Unclear	No	No	Unclear
Li and Yan 2009 [25]	Unclear	Unclear	Unclear	Unclear	No	No	Unclear
Fu et al. 2010 [26]	Unclear	Unclear	Unclear	Unclear	No	No	Unclear
Zhang et al. 2011 [27]	Unclear	Unclear	Unclear	Unclear	No	No	Unclear
Liu and Li 2011 [28]	Unclear	Unclear	Unclear	Unclear	No	No	Unclear
